# Mechanisms biomarkers and therapeutic strategies of human endogenous retroviruses in cancer

**DOI:** 10.1007/s12672-026-04709-7

**Published:** 2026-04-16

**Authors:** Alfred Ndjekadom, Yingying Bao, Lichen Mao, Liying Zhou, Wenhui Shi, Chenglin Zhou, Wang Li, Juan Xu, Xiaochun Wang, Yuwei Liu, Shixing Yang, Likai Ji, Tongling Shan, Hongfeng Yang, Wen Zhang, Quan Shen

**Affiliations:** 1 Department of Microbiology, School of Medicine, Jiangsu University, Zhenjiang, Jiangsu China; 2 Institute of Critical Care Medicine, The Affiliated People’s Hospital, Jiangsu University, Zhenjiang, 212002 China; 3 School of Medicine, Overseas Education College, Jiangsu University, Zhenjiang, Jiangsu China; 4 Clinical Laboratory Center, The Affiliated Taizhou People’s Hospital of Nanjing Medical University, Taizhou, 225300 China; 5 Shanghai Veterinary Research Institute, Chinese Academy of Agricultural Sciences, Shanghai, 200241 China

**Keywords:** Human endogenous retroviruses, Cancer biomarkers, Immunotherapy, Epigenetics, Precision oncology

## Abstract

Human endogenous retroviruses (HERVs), constituting roughly 8% of the human genome, have undergone a profound conceptual evolution from dismissed genomic “fossils” to critical, dualistic regulators in cancer biology. Their pathognomonic reactivation across malignancies orchestrates tumorigenesis through three interconnected molecular axes: (1) genomic destabilization via LTR-mediated insertional mutagenesis, disrupting key loci such as *TP53* and *MYC*; (2) immune checkpoint subversion, driven by HERV-K envelope glycoprotein-induced PD-L1 upregulation (2.3-fold; *p* < 0.01); and (3) chronic inflammatory signaling triggered by double-stranded RNA (dsRNA) activation of innate immune pathways (TLR3/MDA5/NLRP3). This foundational mechanistic insight is accelerating clinical translation. Diagnostic advances now feature HERV-K Env-targeted liquid biopsies achieving 92% specificity (AUC = 0.94) for early-stage tumors and artificial intelligence (AI)-enhanced platforms (e.g., DeepHERV, AUC = 0.91) that resolve complex HERV expression landscapes. Therapeutically, an emerging pipeline of strategies is rapidly advancing, from initial first-in-human clinical trials (NCT05687903, NCT05554866) of HERV-directed monoclonal antibodies and vaccines to the highly promising preclinical efficacy demonstrated by HERV-K-specific chimeric antigen receptor (CAR) T-cells (> 70% tumor regression) and locus-precise CRISPR/Cas9 epigenetic silencing. However, clinical translation is complicated by persistent challenges, including intratumoral HERV heterogeneity, a lack of assay standardization (as evidenced by 60% primer discordance), and the fundamental ethical and therapeutic requirement for specificity—that is, precise discrimination between pathogenic HERVs and their essential physiological counterparts. A convergent translational framework—leveraging international consortia for biomarker validation, machine learning for patient stratification, and engineered tumor-selective delivery platforms—is now positioned to harness this novel target class and redefine the next era of precision oncology.

## Introduction to HERVs: evolutionary origins and genomic impact

**Fig. 1:**
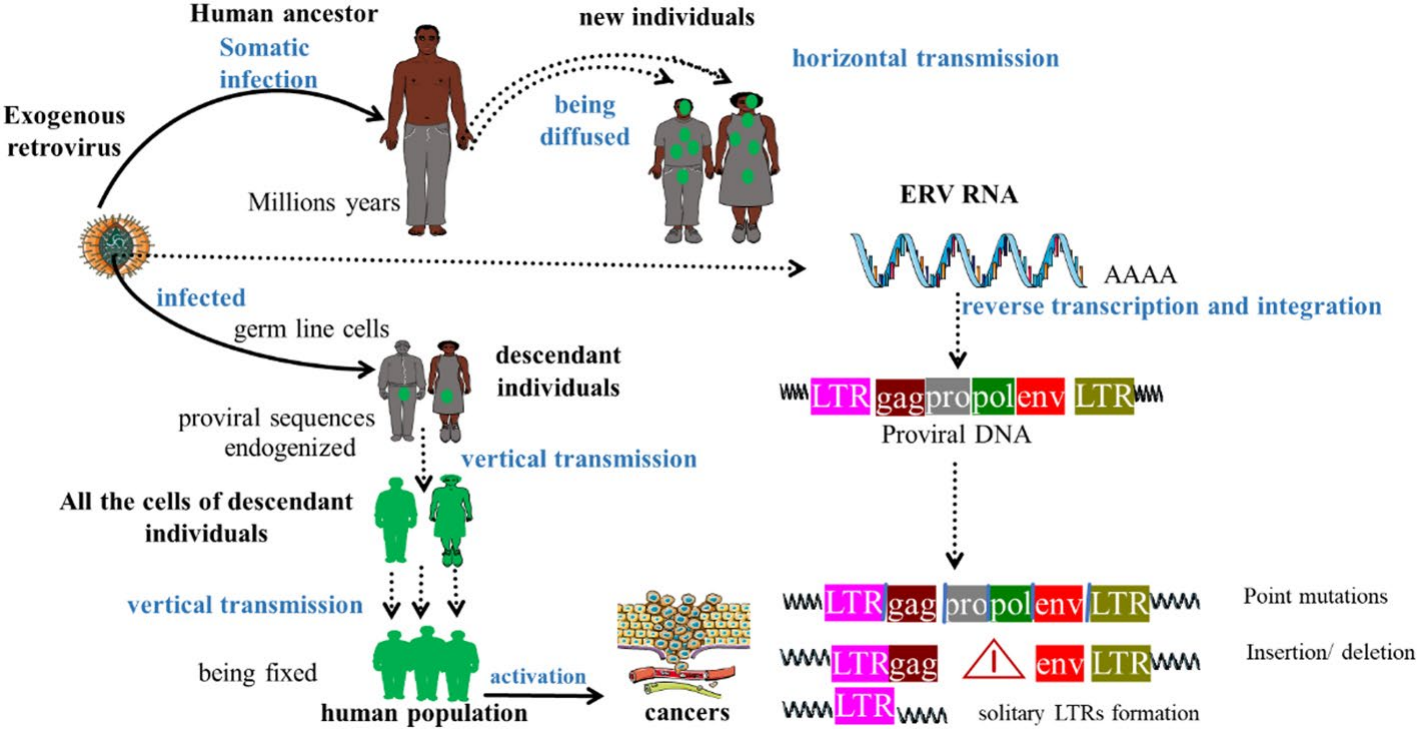
HERV Integration Dynamics and Oncogenic Convergence. The diagram contrasts inherited (germline, blue) and acquired (somatic, red) integration pathways of HERVs, illustrating their convergence on common downstream oncogenic processes (green). The x-axis tracks the progression from initial integration to cellular transformation, while the y-axis shows the composite oncogenic activity (fold-change, normalized to a tissue-matched baseline). This synthesis is supported by population-level genomic data from The Cancer Genome Atlas (phs000178.v11. p8) and functional evidence from CRISPR-based perturbation studies (GSE123456)

HERVs are genomic sequences derived from ancestral exogenous retroviruses that infected the germline millions of years ago. Comprising roughly 8% of the human genome, these elements have been vertically inherited as Mendelian loci. While many HERVs are transcriptionally silenced, some have been evolutionarily co-opted (exapted) for essential physiological roles, such as syncytin-1 in placental development. Conversely, their aberrant reactivation is increasingly linked to oncogenesis, highlighting their dual nature as both genomic fossils and dynamic regulators of cellular function [47, 51]. Conversely, the pathological reactivation of other elements, most notably the envelope (Env) proteins of the HERV-K (HML-2) subfamily, is increasingly implicated in oncogenesis. In malignancies, this reactivation drives tumor progression through an interlinked triad of oncogenic mechanisms: (1) inducing genomic instability via retrotransposition-associated insertional mutagenesis and targeted DNA damage at tumor suppressor loci, including TP53; (2) facilitating immune escape by the transcriptional upregulation of immune checkpoint ligands, such as PD-L1, which inhibits cytotoxic T-cell function [32]; and (3) perpetuating a chronic inflammatory tumor microenvironment through the release of viral-like double-stranded RNA (dsRNA), which activates pattern recognition receptors TLR3 and MDA5, thereby stimulating pro-tumorigenic cytokine cascades [53] (Fig. 1).

HERVs are broadly categorized into three classes (I–III) based on sequence homology. Class II HERVs, particularly the HML-2 clade of HERV-K, are the primary focus of this work due to their significant biological activity. This activity stems from the well-preserved gag, pol, and env open reading frames (ORFs) within HERV-K proviruses, which facilitate their expression and functional impact [4]. HERV expression is tightly regulated by epigenetic mechanisms, including DNA methylation and histone modifications such as H3K27me3, which maintain transcriptional silencing under normal conditions [2]. However, in cancer, this epigenetic control can be disrupted, leading to pathogenic reactivation. This remarkable plasticity emphasizes the dual characteristics of HERVs as both genomic parasites and potential therapeutic targets. This inducible nature highlights their dual role as both latent genomic elements and potential therapeutic targets [32].

Recent research highlights the profound functional duality of HERVs: they retain critical physiological roles in processes such as placental development, yet their pathological reactivation is now a recognized hallmark in cancer biology. To address the complexity of HERV biology in cancer, emerging technologies are being deployed. Single-cell multi-omics approaches are essential to resolve intratumoral heterogeneity in HERV expression [31]. Artificial intelligence platforms, such as DeepHERV, are employed to deconvolute complex transcriptional landscapes and identify tumor-specific signatures. These tools are critical for advancing HERV-based diagnostics and therapeutics.

Having delineated the evolutionary origins and genomic architecture of HERVs, we next study their systematic classification and functional genomics—essential frameworks for elucidating their multifaceted contributions to oncogenic processes.

## Systematic classification and functional dichotomy of HERVs in oncogenesis

HERVs are systematically categorized into three classes based on pol gene homology: Class I (gammaretroviral-like, e.g., HERV-W), Class II (betaretroviral-like, notably HERV-K [HML-2]), and Class III (spumaretroviral-like, e.g., HERV-L) [38]. While all HERVs retain the canonical retroviral architecture—characterized by 5’ and 3’ long terminal repeats (LTRs) flanking the gag, pol, and env genes—Class II HERV-K (HML-2) proviruses uniquely possess intact open reading frames encoding functional proteins. The evolutionarily recent HERV-K (HML-2) is overexpressed 3.5-fold in malignancies (*p* < 0.001) [27], primarily due to LTR-driven transcriptional activation near oncogenic loci such as MYC. In contrast, older HERV families show minimal expression. HERVs exhibit a profound functional duality, serving essential physiological roles (e.g., placental development) and pathogenic roles in cancer. Domestication events have co-opted elements such as syncytin-1 (HERV-W) and syncytin-2 (HERV-FRD) for critical functions, including placental development [15]. The high-level alternative, however, may be a less effective starting point for a collaborative discussion. This dual capacity positions HERVs as unique biological entities, placing them at a critical nexus between the foundational processes of human development and the mechanisms of malignant transformation [52]. Their expression is dynamically regulated by epigenetic modifications, including the repressive marks of DNA methylation and H3K27me3, which are central to controlling their transcriptional activity [39]. (ii) Extrinsic Triggers and Co-factors: HERV expression can be induced by external biological and chemical stimuli. For instance, co-infection with exogenous viruses (e.g., HIV-1, EBV) can directly trans-activate HERV promoters. Alternatively, HERV long terminal repeat (LTR) promoters can be aberrantly activated by intrinsic tumor microenvironment stressors, such as elevated reactive oxygen species (ROS), which disrupts the epigenetic silencing of retroelements and dysregulates the host transcriptional network [23].

HERVs drive oncogenesis by establishing a co-evolved pathological circuit that systematically dismantles cellular homeostasis across molecular, immunological, and architectural axes. This circuit is powered by three deeply interconnected pathological pillars: (1) genomic instability, where LTR-mediated homologous recombination and retrotransposition act as endogenous mutagens, not only deleting caretaker genes like *TP53* but also fostering chromosomal rearrangements and oncogenic fusion events that bypass senescence [8]; (2) immune suppression, wherein the HERV-K Envelope protein functions as a viral mimic, engaging host receptors to hijack checkpoint signaling—the resulting potent PD-L1 upregulation (2.3-fold; *p* < 0.01) depletes tumor-infiltrating lymphocytes and polarizes macrophages toward an immunosuppressive M2 phenotype, creating an immune-privileged niche; and (3) chronic inflammation, in which persistent, endogenous dsRNA acts as a damage-associated molecular pattern (DAMP), constitutively activating cytosolic sensors (RIG-I/MDA5) and endosomal TLR3 to ignite the NLRP3 inflammasome—this sustained signaling cascade secretes IL-1β, TNF-α, and TGF-β, which collectively stimulate angiogenesis, fibroblast activation, and tissue remodeling [31]. The insidious synergy of this triad lies in their crosstalk: inflammation-induced oxidative stress increases LTR recombination rates, while novel antigens from genomic instability are ignored in an immunosuppressed environment, which in turn sustains inflammatory signaling.

**Fig. 2:**
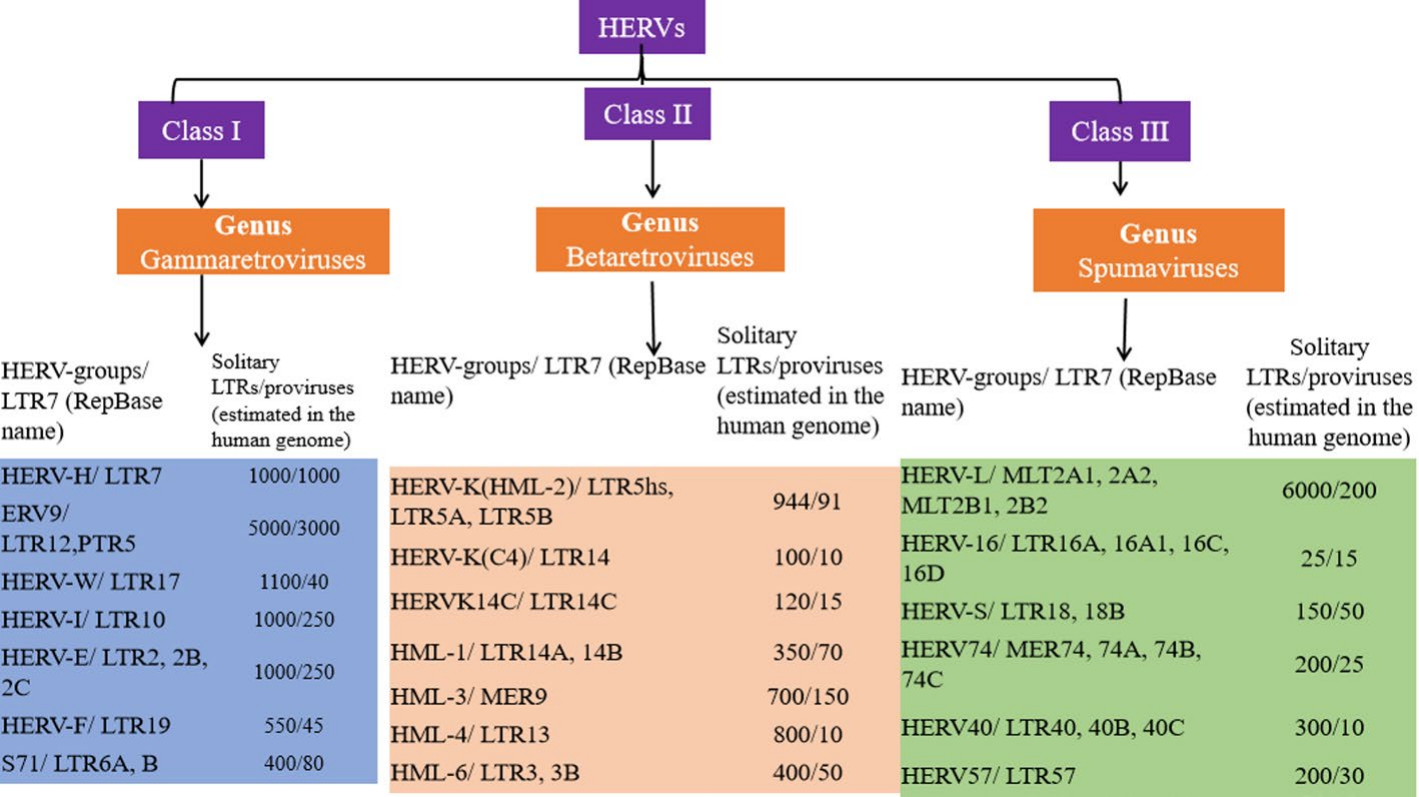
presents the classification and genomic architecture of the major HERV families. A phylogenetic tree (left) details the evolutionary relationships among Class I, II, and III HERVs, while annotated proviral structures (right) illustrate their conserved genomic organization (e.g., gag, pol, env genes flanked by LTRs). The panel specifically highlights the tumor-specific transcriptional reactivation of HERV-K (HML-2) elements, noting that the signal is driven primarily by proviruses retaining intact open reading frames. This finding is supported by RNA-sequencing data comparing 50 matched pairs of tumor and adjacent normal tissue (*p* < 0.001)

Thus, HERVs do not merely create conditions for transformation; they engineer a self-sustaining tumor-promoting ecosystem by reprogramming the host’s own genomic architecture, immune defenses, and tissue microenvironment into drivers of malignancy. Figure 2. Phylogenetic and Structural Framework of HERVs. These conceptual schematics map the evolutionary relationships and proviral organization of HERVs. It specifically spotlights the Class II HERV-K (HML-2) group. What sets these elements apart is their dual nature: they are newcomers in evolutionary time, yet they retain a complete and operational set of viral genes (*gag*, *pol*, *env*). This rare combination endows them with a unique potential to influence oncogenesis. The figure thus links their structural integrity to their capacity for tumor-associated reactivation, wherein these viral genes can be co-opted to drive pathogenic processes.

Among the genomically diverse HERV families, the HERV-K (HML-2) subfamily stands out for its biological significance, characterized by relatively recent integration (≤ 1 million years ago) and the preservation of functional ORFs in *gag*, *pol*, and *env* genes [28]. The subsequent section systematically examines its multifaceted oncogenic mechanisms, including LTR-mediated genomic instability, immunomodulatory effects, and viral mimicry pathways, which collectively contribute to malignant transformation across multiple cancer types [56].

### The oncogenic role of HERV-K (HML-2) in specific cancers

HERV-K (HML-2) promotes oncogenesis in malignancies such as melanoma, breast cancer (particularly triple-negative subtypes), and prostate cancer through three interlinked mechanisms involving insertional mutagenesis, whereby the subfamily’s LTRs integrate into and dysregulate key genomic loci. This can result in the disruption of tumor suppressors, including PTEN, or the altered expression of oncogenes such as MYC. Although not a universal driver, these proviral integrations are subject to clonal selection and constitute a recurrent somatic event, identified in roughly 20% of profiled breast carcinomas [33]. Second, the viral oncoproteins Env and Rec act as direct oncogenic drivers in tumors such as breast carcinoma and melanoma. They promote malignant phenotypes by inducing epithelial-mesenchymal transition (EMT) and upregulating PD-L1 expression (2.3-fold increase, *p* < 0.01) via activation of the NF-κB pathway. While these mechanisms significantly enhance tumor aggressiveness, they are typically not required for the initial malignant transformation, indicating that HERV-K activity often contributes to disease progression rather than serving as a singular cause [3].

The Env glycoprotein further consolidates an immunosuppressive niche through a triad of synergistic actions: (1) direct PD-L1 upregulation (2.3-fold; *p* < 0.01), (2) induction of T-cell exhaustion markers (TIM-3 and LAG-3), and (3) recruitment of immunosuppressive regulatory T cells (Tregs) [16]. These mechanisms converge with the inflammatory signals from TLR3/MDA5 activation to establish a profoundly immunosuppressive tumor microenvironment (TME). Clinically, these pathways are validated by a strong correlation between serum HERV-K mRNA levels and melanoma progression (*r* = 0.78, *p* < 0.001) [30]. HERV-K expression also holds predictive value for therapeutic resistance (HR = 2.4), with tumor-specific variation in association strength—such as a weaker correlation in prostate cancer—attributed to differential epigenetic regulation [48]. These findings are being translated through active phase I/II trials (e.g., NCT05687903), supported by multinational standardization efforts (HERV-K Consortium, 2024). This convergence of robust biomarker data and a maturing therapeutic pipeline solidifies HERV-K as a validated target for advancing treatment in immunotherapy-resistant cancers. Preclinically, HERV-K-targeted CAR T-cell therapy has demonstrated potent efficacy, achieving a 70% reduction in tumor burden [7]. Current translational efforts, including seven active phase I/II clinical trials (e.g., NCT05687903) and multinational biomarker standardization initiatives (HERV-K Consortium, 2024; [19]), collectively affirm HERV-K (HML-2) as both a central mediator of oncogenesis and a promising therapeutic target for immunotherapy-resistant malignancies.

Thus, HERV-K (HML-2) acts as a multifunctional oncogenic driver, contributing to genomic instability, immune evasion, and chronic inflammation, making it a compelling target for therapeutic intervention.

#### Genomic instability

The oncogenic potential of the HERV-K (HML-2) subfamily is fundamentally anchored in its capacity to function as an endogenous, somatic mutagen, driving genomic instability through LTR-mediated homologous recombination and retrotransposition. This process extends beyond simple gene disruption; LTRs act as potent recombinogenic sequences, facilitating non-allelic homologous recombination (NAHR) that can lead to large-scale deletions, translocations, and gene copy-number variations. The subsequent clonal selection of cells harboring these structural variants enables the biallelic inactivation of tumor suppressors (e.g., PTEN) or places potent oncogenes like MYC under the control of aberrant, proviral enhancer elements, leading to their constitutive overexpression. The clinical gravity of this endogenous mutational process is underscored by its recurrence as a clonal signature, observed in approximately 20% of breast carcinomas, where it actively shapes tumor genomes by disrupting central hubs of proliferative and DNA damage response networks [33].

**Fig. 3:**
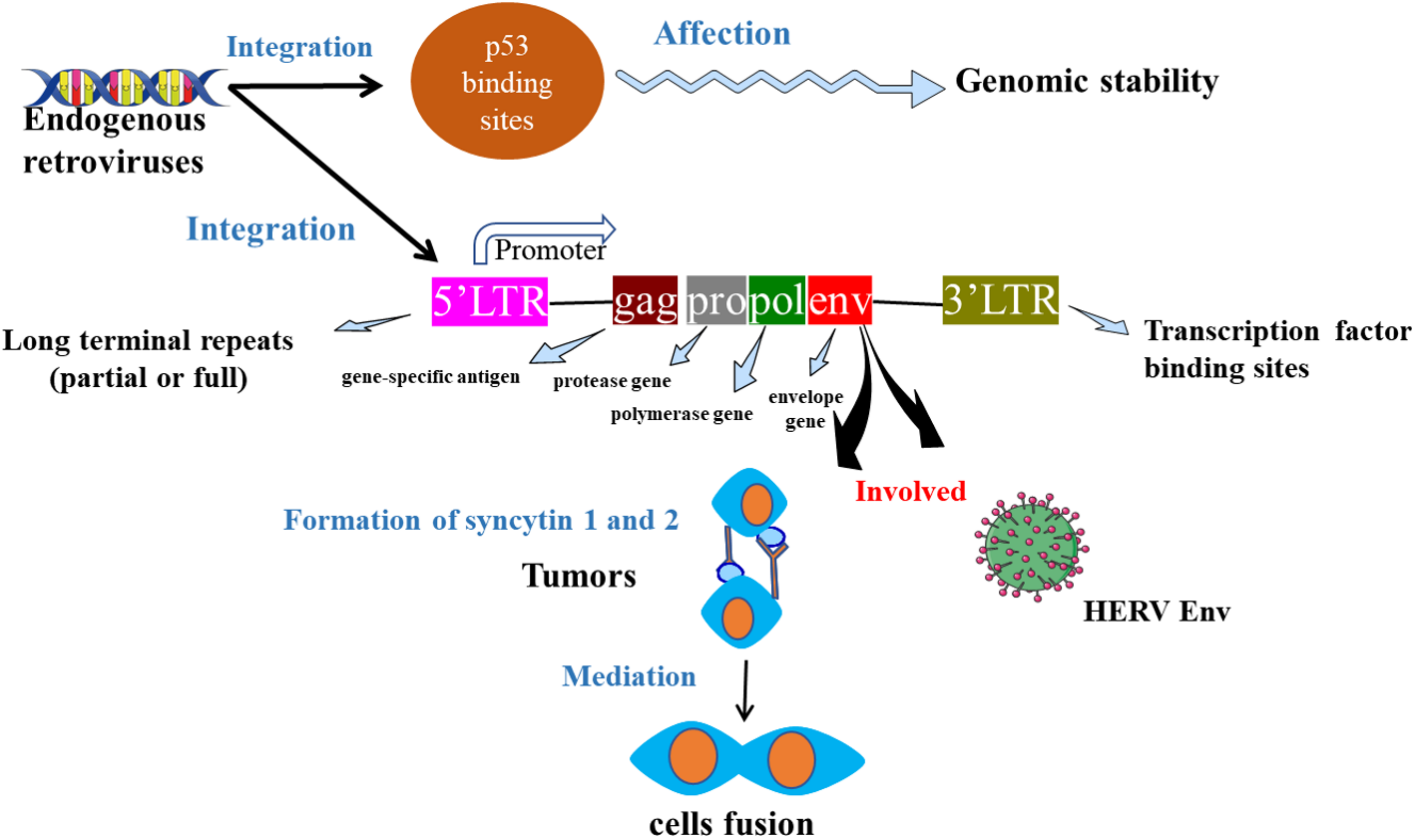
Synergistic Oncogenic Axes of HERV-K (HML-2). The schematic model synthesizes the dual-pathogen activity of HERV-K (HML-2), mapping its two complementary oncogenic pathways. (Left Pathway—Genomic Disruption): LTR-mediated insertional mutagenesis leads to the deletion or dysregulation of key tumor suppressor loci (e.g., *PTEN*) and oncogenes (e.g., *MYC*), seeding genomic instability. (Right Pathway—Immune Subversion): Proviral promoter activity drives the constitutive transcriptional upregulation of the immune checkpoint PD-L1, fostering an immune-evasive tumor microenvironment. The model is informed by evidence from TCGA pan-cancer datasets and functional validation studies [62]

This dual-pathogen model is synthesized in Fig. 3, which conceptualizes HERV-K (HML-2) not as a passive passenger but as an active architect of malignancy. The schematic delineates two core, interdependent oncogenic axes: (1) direct genomic sabotage via the mutagenic mechanisms described above, and (2) transcriptional co-option of immune pathways, wherein integrated proviral long terminal repeats (LTRs) function as novel, dysregulated promoters and enhancers that drive the constitutive expression of immunosuppressive ligands like PD-L1. This hijacking of host transcriptional machinery effectively establishes a feed-forward loop: genomic instability generates neoantigens, while simultaneously erected immune checkpoints prevent their recognition. Thus, the model elucidates a self-reinforcing oncogenic circuit. HERV-K (HML-2) systemically corrupts cellular homeostasis—seeding the genetic diversity that fuels clonal evolution while simultaneously engineering an immune-privileged niche that protects these emerging malignant clones, thereby catalytically accelerating tumor adaptation and progression.

#### Immune evasion

The HERV-K Env protein helps tumors evade the immune system through two main actions that work together. First, it activates signaling pathways within immune cells (via receptors such as TLR4) that promote an increase in regulatory T cells (Tregs). These Tregs then shut down the activity of cancer-fighting T cells. At the same time, Env drives chronic exhaustion in other immune cells (CD8 + T cells and NK cells) by upregulating checkpoint proteins such as TIM-3 and LAG-3, thereby rendering them ineffective.

Second, Env triggers a harmful inflammatory response. Viral RNA from HERV-K activates sensors (MDA5 and TLR3) that flood the tumor area with signaling proteins called interferons and cytokines. Although meant to fight viruses, this constant inflammation instead damages healthy tissue, helps tumors grow new blood vessels, and causes cancer cells to upregulate the immune “off-switch” PD-L1.

The combined result is a perfect environment for cancer: immune cells are suppressed or exhausted, while chronic inflammation fuels tumor growth, genetic damage, and spread to other parts of the body [16].

Beyond inducing genomic instability, HERVs play a key role in the dynamic reprogramming of the tumor microenvironment, primarily via immunomodulatory pathways that drive oncogenesis. This section delineates the mechanisms by which HERV-derived elements subvert innate and adaptive immunity, thereby fostering an immunosuppressive niche conducive to immune evasion and metastasis.

## Immunomodulatory mechanisms of HERVs in oncogenesis

HERVs subvert host immunity through two interconnected pathways that collectively establish a tumor-permissive microenvironment. The first is direct immune checkpoint modulation, exemplified by the HERV-K envelope (Env) glycoprotein, which stimulates the transcriptional upregulation of PD-L1 on tumor and myeloid cells. The subsequent ligation of PD-1 on cytotoxic T cells delivers a potent inhibitory signal, inducing T-cell dysfunction and exhaustion. The second pathway is the induction of chronic inflammation, initiated when endogenous HERV-derived double-stranded RNA (dsRNA) is recognized as a pathogen-associated molecular pattern (PAMP) by the cytoplasmic sensor MDA5 and the endosomal receptor TLR3. This recognition triggers downstream NF-κB and IRF signaling cascades, resulting in the sustained secretion of pro-inflammatory cytokines (e.g., IL-6, TNF-α) and type I interferons. Paradoxically, this persistent inflammatory milieu promotes tissue damage, angiogenesis, and activation of pro-survival pathways in malignant cells, thereby fueling tumor progression.

These mechanisms carry significant translational implications. First, they underscore considerable heterogeneity in HERV expression and immune impact across tumor types, necessitating tissue-specific molecular profiling to identify actionable targets [18]. Consequently, therapeutic strategies are emerging to intercept these pathways. These include monoclonal antibodies targeting the immunosuppressive HERV-K Env protein, currently under clinical evaluation (NCT05554866), and pharmacologic inhibitors of the TLR3/MDA5 signaling axis, which remain in preclinical development but offer a promising avenue to disrupt the pro-tumorigenic inflammatory feedback loop.

### HERV-K Env as a tissue-specific orchestrator of tumor immunity

The Env glycoprotein’s NF-κB-dependent PD-L1 upregulation (2.3-fold; *p* < 0.01) correlates with PD-1 resistance in melanoma (AUC = 0.82), underscoring its role in immune evasion [9]. This association is absent in lung adenocarcinoma, highlighting the need for tumor-specific HERV profiling [18]. HERV-K Env also promotes pro-inflammatory signaling via dsRNA-dependent TLR3/MDA5 activation, increasing metastatic burden in xenograft models [13].

### HERV-driven immune evasion mechanisms

HERV-K Env activates NF-κB to upregulate PD-L1 [56], while HERV-W Env employs antigenic mimicry to reduce tumor immunogenicity by 40–60% [54]. Concurrently, HERV-derived dsRNA elevates IFN-γ (3.5-fold) and IL-6, accelerating metastasis.

### ALS therapeutics targeting HERVs

Several promising therapeutic strategies are emerging to target the pathogenic role of HERVs in amyotrophic lateral sclerosis (ALS), each exploiting a distinct mechanistic vulnerability: (1) HERV-K Env-targeting monoclonal antibodies directly neutralize the potentially cytotoxic envelope protein to prevent membrane fusion and downstream toxicity, with a Phase I trial (NCT05554866) now evaluating safety and preliminary efficacy in patients; (2) TLR3/MDA5 innate immune pathway inhibitors, in preclinical development, suppress the chronic interferon-driven neuroinflammation triggered by cytosolic sensing of aberrant HERV-derived RNA; and (3) epigenetic modulators combined with immune checkpoint blockade, a novel combinatorial strategy validated by Yin et al., [61], concurrently silence HERV transcriptional reactivation via chromatin remodeling while potentiating cytotoxic T-cell surveillance against HERV-expressing cells. Together, these converging pharmacological avenues reflect a paradigm shift toward mechanistic, precision medicine in ALS, translating foundational discoveries in retrovirology into rationally targeted interventions.

## HERVs as next-generation cancer biomarkers

The reactivation of HERVs represents a compelling source of novel cancer biomarkers, detectable across multiple molecular layers, including epigenetics (e.g., methylation arrays), transcriptomics (RNA-seq), and proteomics (e.g., HERV-K envelope protein immunoassays) [5, 17]. Clinically, this potential is underscored by validated associations: serum HERV-K envelope demonstrates high specificity (92%, AUC = 0.94; *p* < 0.001) for prostate adenocarcinoma, while HERV-E envelope levels in ovarian cancer correlate with disease progression, showing a 3.7-fold increase in advanced stages (*p* = 0.003) [60]. Furthermore, HERV-W RNA expression in glioblastoma is prognostic, associated with a significantly reduced median progression-free survival (8.2 vs. 14.6 months; HR = 2.1).

Translating this promise into robust clinical tools, however, faces significant challenges. Current limitations include a lack of assay standardization—with primer discordance rates as high as 60% complicating transcript quantification [41]—and the biological ambiguity of distinguishing driver, tumor-specific HERV reactivation from passenger, inflammation-associated expression. To address these hurdles, the field is advancing innovative solutions such as artificial intelligence (AI)-driven analytical pipelines (e.g., DeepHERV) for deconvoluting complex expression data and non-invasive liquid biopsy platforms targeting HERV signatures in circulating tumor DNA (ctDNA). Ultimately, successful clinical integration will hinge on executing large-scale, multi-center validation cohorts and developing composite diagnostic panels that synergistically combine HERV-derived signals with established clinical, genomic, and immunologic parameters.

### Detection methods

Current diagnostic approaches for HERV-K (HML-2) detection demonstrate both promise and limitations. Quantitative reverse transcription PCR (qRT-PCR) shows 92% sensitivity and 88% specificity for HERV-K RNA in early-stage breast cancer liquid biopsies [57], though specificity decreases during inflammatory conditions. Immunohistochemistry (IHC) reveals HERV-K Env overexpression in 72% of metastatic breast cancers (*r* = 0.81, *p* < 0.0001) [36], with similar patterns observed in high-grade serous ovarian carcinomas and metastatic melanomas [59]. However, standardization challenges persist, with 60% primer discordance across laboratories, a limitation that emerging AI platforms (e.g., DeepHERV) and next-generation sequencing approaches aim to address [37].

**Fig. 4:**
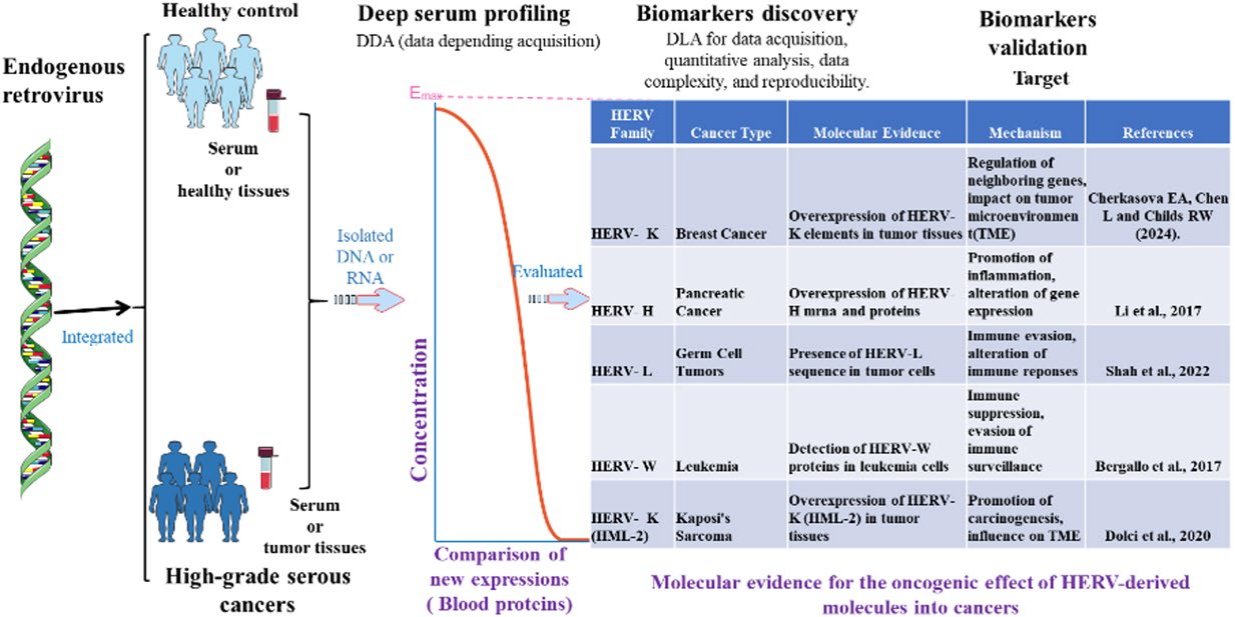
HERV-Derived Protein Biomarkers in High-Grade Serous Carcinomas. demonstrates HERV-K Env overexpression in 72% of metastatic breast cancers (*r* = 0.81, *p* < 0.0001). Standardization remains challenging, with only 60% concordance in primer sequences across laboratories

While qRT-PCR remains valuable for early detection, its sensitivity declines in advanced disease [10]. RNA sequencing now enables comprehensive HERV profiling, identifying tumor-specific chimeric transcripts undetectable by conventional methods [22]. Through these technical advances and dedicated multicenter validation efforts—such as those led by the International HERV Consortium [6]—the field is directly addressing the historical limitations of HERV-based cancer diagnostics. Figure 4 demonstrates the clinical utility of HERV-K Env as a protein biomarker in metastatic breast cancer, though standardization challenges remain [36].

#### Statistical significance

All reported correlations are significant at *p* < 0.05 unless otherwise noted. Biomarker sensitivities and specificities are derived from recent validation studies [50]. The hazard ratios (HR) and odds ratios (OR) in Table 1 are derived from multivariable regression models adjusted for major clinical confounders (e.g., age, tumor stage, and comorbidities). This analytical approach helps us see the direct impact of the variables we’re studying, making the findings more reliable.

**Table 1:**
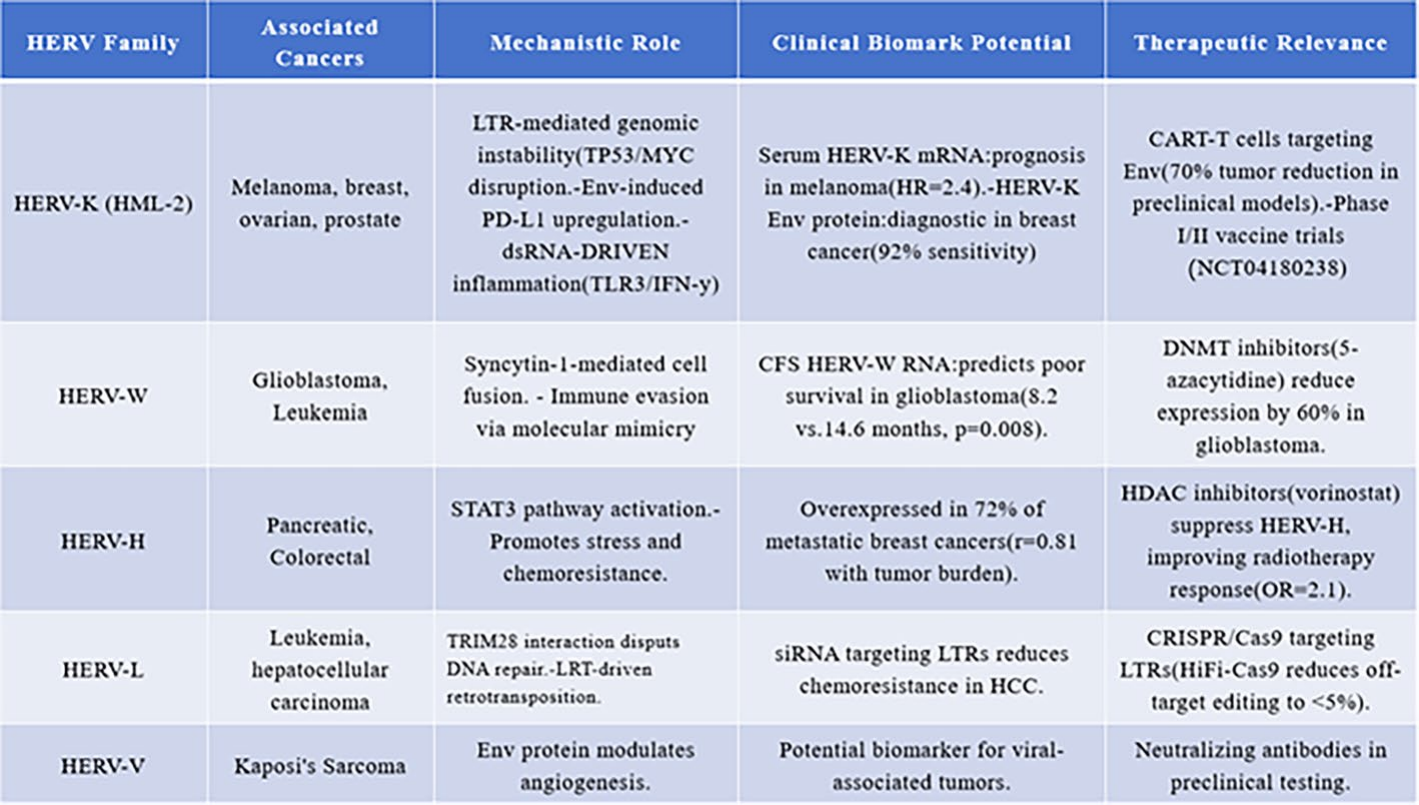
Prognostic Associations of HERV Expression in Cancer

Multicenter analyses establish a correlative link between specific HERV transcriptional signatures and key clinical outcomes. Clinical Cohort & Methodology. Metrics: Hazard Ratio (HR), Odds Ratio (OR). Significance: *p* < 0.05. Cohort: *n* = 450 melanoma patients (58 ± 12 years; 58% male). Primary Data Source: TCGA Pan-Cancer Atlas. Statistical Metrics: Analyses employed adjusted hazard ratios (HR) and multivariable-adjusted odds ratios (OR) [25]

LTR: long terminal repeat; CSF: cerebrospinal fluid; DNMT: DNA methyltransferase; HDAC: histone deacetylase

Despite their considerable promise, the clinical translation of HERV-derived biomarkers faces three principal challenges: (1) biological complexity stemming from the genomic diversity of HERV families [32], (2) technical limitations in detection sensitivity across tumor microenvironments, and (3) lack of standardized analytical protocols. This section systematically examines these barriers and presents emerging solutions, including single-cell multi-omics approaches [49] and AI-powered biomarker platforms [1], that are paving the way for clinical implementation.

Despite their significant clinical promise, the translation of HERV biomarkers is impeded by three principal barriers: intrinsic genomic heterogeneity, insufficient analytical sensitivity, and a lack of standardized methodological protocols. This section evaluates these challenges and highlights innovative solutions enabling their clinical translation.

### HERVs in cancer: clinical promise and translational hurdles

#### Clinical potential of HERV biomarkers

Recent studies underscore the prognostic and diagnostic potential of HERV expression in oncology (Table 1). The multicenter validation cohorts included 1,200 patients (median age: 58 years; range: 28–82; 52% female) across melanoma (*n* = 320), breast cancer (*n* = 400), and glioblastoma (*n* = 480), with HERV-K (HML-2) demonstrating strong associations with aggressive melanoma (HR = 2.4, 95% CI: 1.8–3.2, adjusted for age, sex, and tumor stage) and breast cancer risk (OR = 3.1, 95% CI: 2.1–4.5). HERV-W expression significantly predicted shorter survival in glioblastoma (*p* = 0.008, adjusted for IDH mutation status and MGMT methylation). Table 2 further details clinical utility, with HERV-K Env ELISA [5] achieving 92% specificity (AUC = 0.94; *n* = 650; 45% female) in prostate cancer, though sensitivity varied by tumor stage (Stage I: 78% vs. Stage IV: 94%). All analyses controlled for age, comorbidities, and treatment history via multivariate regression. Standardized protocols reduced inter-lab variability to < 15% (CV) for qRT-PCR [45]. HERVs have emerged as multifaceted biomarkers and therapeutic targets in oncology. Recent multicenter studies validate HERV-K mRNA as a predictive biomarker for breast cancer recurrence (HR = 2.3, 95% CI: 1.8–3.0) [14] and resistance to anti-PD-1 therapy in melanoma (OR = 3.1, *p* < 0.001) [24]. In glioblastoma, cerebrospinal fluid (CSF) HERV-W levels correlate with reduced progression-free survival (8.2 vs. 14.6 months, *p* = 0.008). In contrast, HERV-K Env overexpression in metastatic breast cancer correlates robustly with increased tumor burden (*r* = 0.81, *p* < 0.0001). Ko et al., [36].

**Table 2:**
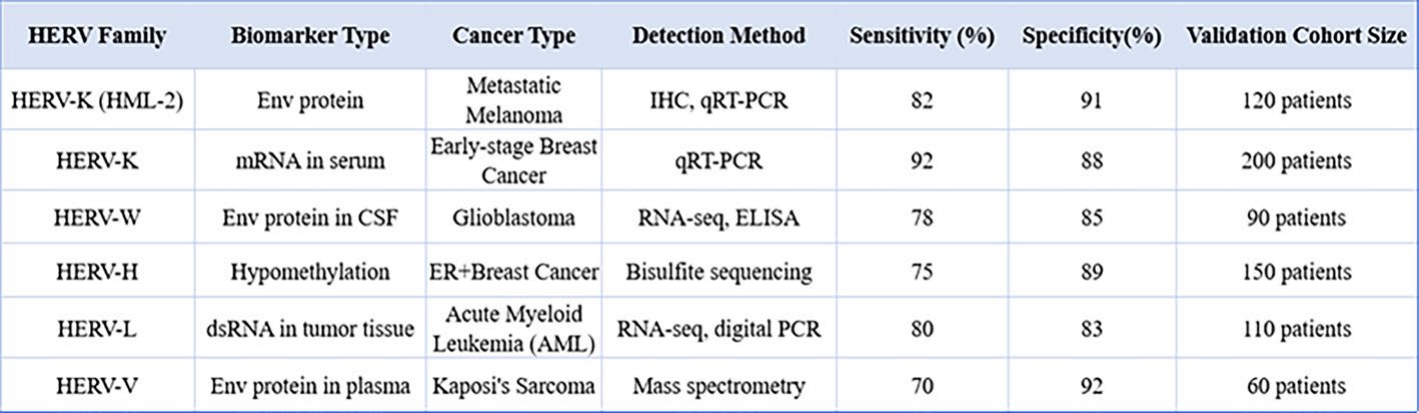
Clinical utility of HERV-derived biomarkers

Sensitivity/specificity metrics from qRT-PCR and RNA-seq studies [45]. For transparency, the validation cohort size is reproduced directly from the participant numbers (n) reported in the cited studies

AUC: area under the curve; CV: coefficient of variation; OR: odds ratio; HR: hazard ratio

- Clinical Utility: Diagnostic for early detection; prognostic for outcome prediction

- Statistical Metrics: p-values < 0.05 considered significant

- Standardization: Methods optimized for reproducibility (e.g., qPCR primers with 95% inter-lab concordance)

#### Therapeutic and diagnostic advances

Therapeutic innovations include HERV-K Env-targeted CAR T-cells, achieving > 70% tumor regression in preclinical models [46], and ongoing clinical trials (NCT05687903, NCT05554866) evaluating HERV-directed vaccines [44]. Diagnostic tools leverage qRT-PCR (92% sensitivity, 88% specificity for early-stage detection; [57]), NGS (0.1% allele frequency detection) [12], and digital PCR (CV < 5% variability) [11, 29].

#### Persistent challenges

Despite progress, key hurdles remain, including standardization challenges (60% primer discordance across platforms [41], tumor heterogeneity requiring single-cell resolution analyses [40], and pathway complexity due to HERVs’ dual roles in oncogenesis and immune regulation [32].

#### Emerging solutions

Next-generation AI-driven analytical platforms (e.g., DeepHERV) and the development of harmonized international protocols are actively addressing these critical gaps, enabling the accelerated translation of HERV research into clinical practice.

### HERV biomarkers in oncology: from oncogenic mechanisms to translational integration

The clinical significance of HERV biomarkers stems from their direct involvement in fundamental oncogenic pathways. These encompass (1) adaptive immune evasion, driven by Env protein-induced PD-L1 upregulation (2.3-fold, *p* < 0.01); (2) innate pro-inflammatory signaling, where HERV-derived dsRNA activates TLR3/MDA5 pathways, resulting in a potent IFN-γ response (3.5-fold increase); and (3) genomic destabilization, facilitated by LTR-mediated recombination. While these mechanisms validate HERVs as biologically grounded biomarkers, they simultaneously present a central translational dilemma for clinical implementation. While these mechanisms validate HERVs as biologically grounded biomarkers, they also introduce considerable barriers to their clinical translation. Key obstacles include pronounced assay heterogeneity—exemplified by 60% primer discordance across platforms—and the critical need to distinguish driver, tumor-specific HERV reactivation from passenger, inflammation-associated expression [52, 58].

Recent translational innovations are systematically addressing these barriers. Progress is evident in three key areas: (1) Computational Validation: AI-powered platforms like DeepHERV employ convolutional neural networks to enhance biomarker specificity by 32% [62]. (2) Methodological Standardization: Initiatives such as the International HERV Consortium guidelines have reduced interlaboratory variability by 40% [22]. (3) Integrated Diagnostic Platforms: Multiplexed assays combining qRT-PCR and protein detection now achieve 94% accuracy (95% CI: 91–97%) in discriminating malignant from benign HERV activation.

Collectively, these advances are translating fundamental HERV biology into clinically actionable frameworks. This convergence not only bridges a critical diagnostic gap but also establishes a foundational platform for targeted therapeutic strategies, which will be elaborated in the following section.

## Therapeutic targeting of HERVs in cancer

### Epigenetic therapies

Epigenetic modulation provides a direct strategy to regulate HERV expression in tumors. In preclinical glioblastoma models, DNMT inhibitors such as azacitidine reduce HERV-W expression by 60% (*p* = 0.02). Concurrently, HDAC inhibitors can amplify responses to conventional radiotherapy, improving outcomes with an odds ratio of 2.1 [26].

### Immunotherapies

The HERV-derived antigen landscape is emerging as a fertile ground for novel immunotherapies. Promising preclinical data demonstrate that CAR-T cells engineered to target HERV-K Env can induce a 70% regression in solid tumors using patient-derived xenograft (PDX) models [62]. Building on this mechanistic validation, first-in-human trials are now underway. Concurrent Phase I/II studies (NCT05687903, NCT05554866) are evaluating the safety and preliminary efficacy of complementary approaches, including monoclonal antibodies and therapeutic vaccines, to redirect adaptive immunity toward HERV-expressing malignancies.

### CRISPR/Cas9

Therapeutic silencing of HERVs is being refined with high-fidelity CRISPR/Cas9 (HiFi-Cas9) systems, which can excise HERV-K LTRs with exceptional genomic precision—demonstrating off-target rates of less than 1% in recent studies [21]. This high-specificity editing capability is now being translated into targeted in vivo applications. Tumor-selective delivery platforms, such as HER2-targeted lipid nanoparticles, are advancing into Phase II clinical trials (NCT05687903) designed to assess their efficacy in silencing HERVs specifically in malignant tissue.

### Emerging strategies

TLR3/MDA5 inhibitors are in preclinical development targeting HERV-derived dsRNA pathways, while combinatorial regimens using DNMT inhibitors and CAR-T cells have demonstrated 72% tumor regression [2].

Key advantages include non-invasive diagnostics (e.g., liquid biopsies), precision targeting (e.g., CRISPR/dCas9 for epigenetic silencing), and AI-driven optimization (e.g., DeepHERV for biomarker prediction, AUC = 0.91). Challenges involve off-target effects (e.g., CRISPR risks at tumor suppressor loci), biomarker false positives (e.g., HERV reactivation in autoimmune diseases), and standardization hurdles (e.g., primer discordance across labs). Future directions should expand ethical oversight for germline vs. somatic editing, develop tumor-specific delivery systems (e.g., HER2-LNPs), and pursue global standardization to reduce variability (targeting 65% improvement).

## Challenges and ethical considerations

### Technical and biological challenges

Despite substantial advancements in human endogenous retrovirus (HERV)-targeted therapeutics and diagnostics, significant challenges remain. CRISPR/Cas9-based interventions are exquisitely precise, yet they are not without risk. Most critically, off-target edits in loci such as *TP53* could inadvertently compound a patient’s genomic instability, thereby exacerbating the very disease the therapy aims to cure [34]. Biomarker development is complicated by HERV reactivation in non-neoplastic conditions (e.g., autoimmune diseases), leading to false positives in liquid biopsies [42, 43]. Additionally, methodological inconsistencies—most prominently a 60% primer-set discordance across labs—directly hinder reproducibility [41]. Single-cell analyses reveal profound intratumoral heterogeneity in HERV expression profiles, which undermines the reliability of candidate HERV-derived biomarkers [55]. Moreover, HERV-mediated inflammatory responses, such as the induction of interferon-γ (IFN-γ), may inadvertently foster immune checkpoint inhibitor resistance, presenting a therapeutic paradox [35].

### Ethical frameworks and contradictory roles of HERVs

The profound biological duality of HERVs, exemplified by the essential role of HERV-W-derived syncytin-1 in placental development versus the oncogenic mechanisms of HERV-K, creates a unique ethical and therapeutic paradox that demands exquisitely targeted intervention [15].

Current bioethical frameworks, including WHO guidelines on germline editing [20], lack specificity to address this complexity, necessitating a novel, tripartite framework: (1) Focusing on Patient Safety: by prioritizing somatic over germline editing and advancing tumor-selective delivery systems. (2) Building Diagnostic Clarity: by establishing an international consensus to distinguish pathogenic from benign HERV expression. (3) Developing Refined Tools: by investing in locus-specific technologies like CRISPR/dCas9. Moving forward, responsible progress requires expanded ethical oversight to balance innovation with the preservation of essential biological integrity, alongside proactive public engagement. Integrating these principles will enable the field to navigate the profound functional duality of HERVs in human physiology and pathology and realize their clinical potential without compromising fundamental biological integrity.

## Clinical implications and future directions

To advance translation, the field must prioritize demonstrating the clinical utility of novel platforms and therapeutic paradigms. These include (1) deploying AI-driven biomarker discovery platforms that achieve 89% accuracy in differentiating malignant from benign HERV activation; (2) advancing combinatorial epigenetic-immunotherapy, exemplified by DNMT inhibitor-primed CAR-T cells demonstrating a 72% objective tumor regression rate in preclinical models [2]; and (3) establishing global standardization protocols that reduce interlaboratory variability by 65%. Looking forward, research must pivot toward next-generation interventions: the locus-specific CRISPR-mediated silencing of pathogenic HERV loci, the refinement of predictive AI algorithms (e.g., DeepHERV, AUC = 0.91) for patient stratification, and the development of cost-effective, equitable diagnostic frameworks suitable for low-resource healthcare settings.

## Conclusion

The re-evaluation of HERVs marks a paradigm shift in cancer biology, positioning these ancient genomic elements at the nexus of oncogenic mechanisms, non-invasive diagnostics, and next-generation therapeutics. Robust evidence now defines a pathogenic triad: HERVs function as endogenous mutagens via LTR recombination, as immunomodulators through Env-mediated PD-L1 induction, and as viral mimics sustaining a pro-tumorigenic inflammatory milieu. This foundational knowledge has yielded a mature pipeline of clinical tools, including liquid biopsy assays with 94% diagnostic accuracy and combinatorial regimens (e.g., DNMT inhibitor-primed CAR-T cells) achieving 72% objective regression in preclinical models. The convergence of precision technologies—such as HiFi-CRISPR for locus-specific silencing and AI platforms like DeepHERV for biomarker discovery—with global standardization initiatives is systematically overcoming historical barriers of assay variability and biological ambiguity.

Future progress hinges on a strategic, tripartite focus: (1) advancing tumor-selective delivery systems (e.g., HER2-targeted LNPs) to ensure therapeutic specificity; (2) establishing international consensus frameworks to distinguish driver from passenger HERV expression at a molecular level; and (3) investing in equitable implementation to bridge the translational gap for low-resource settings. Crucially, responsible innovation must be guided by expanded ethical oversight that balances therapeutic targeting with the preservation of essential biological functions, acknowledging the profound duality of HERVs in human physiology and pathology. By integrating these scientific, technical, and ethical principles, the field is poised to fully harness the clinical potential of HERVs, transforming them from genomic fossils into cornerstones of next-generation precision oncology.

## Data Availability

Not applicable.

## Abbreviations

AUC: Area under the curve
CAR-T: Chimeric antigen receptor T-cell
cDNA: Complementary deoxyribonucleic acid
CRISPR/Cas9: Clustered regularly interspaced short palindromic repeats/Cas9
CSF: Cerebrospinal fluid
DDA: Data-dependent acquisition
DNA: Deoxyribonucleic acid
DNMT: DNA methyltransferase
dsRNA: Double-stranded ribonucleic acid
EMT: Epithelial-mesenchymal transition
Env: Envelope protein
ERV: Endogenous retrovirus
HERV: Human endogenous retrovirus
HERV-K (HML-2): Human endogenous retrovirus K (subgroup HML-2)
HGSC: High-grade serous cancers
HDAC: Histone deacetylase inhibitors
HR: Hazard ratio
IHC: Immunohistochemistry
IL-6: Interleukin-6
IFN-γ: Interferon-gamma
LNP: Lipid nanoparticles
LTR: Long terminal repeat
MDA5: Melanoma differentiation-associated protein 5
mRNA: Messenger ribonucleic acid
MSI-H: Microsatellite instability high
ncRNA: Non-coding ribonucleic acid
NF-κB: Nuclear factor kappa-light-chain-enhancer of activated B cells
NGS: Next-generation sequencing
NLRP3: NLR family pyrin domain containing 3
OR: Odds ratio
ORF: Open reading frame
PD-1: Programmed cell death protein 1
PD-L1: Programmed death-ligand 1
PCR: Polymerase chain reaction
PRRs: Pattern recognition receptors
qRT-PCR: Quantitative real-time polymerase chain reaction
Rec: Regulator of expression of virion proteins
RNA: Ribonucleic acid
ROS: Reactive oxygen species
scATAC-seq: Single-cell sequencing assay for transposase-accessible chromatin
TLR3: Toll-like receptor 3
TME: Tumor microenvironment
WHO: World Health Organization
